# Electrochemical detection of extracellular vesicles for early diagnosis: a focus on disease biomarker analysis

**DOI:** 10.20517/evcna.2023.72

**Published:** 2024-04-29

**Authors:** Jintao Zheng, Runzhi Zhou, Bing Wang, Chang He, Shiyao Bai, Haoyang Yan, Jiacheng Yu, Huaiguang Li, Bo Peng, Zhaoli Gao, Xiean Yu, Chenzhong Li, Cheng Jiang, Keying Guo

**Affiliations:** ^1^Biotechnology and Food Engineering, Guangdong Technion-Israel Institute of Technology (GTIIT), Shantou 515063, Guangdong, China.; ^2^Faculty of Biotechnology and Food Engineering, Technion-Israel Institute of Technology (IIT), Haifa 3200003, Israel.; ^3^NMPA Key Laboratory for Bioequivalence Research of Generic Drug Evaluation, Shenzhen Institute for Drug Control, Shenzhen 518057, Guangdong, China.; ^4^School of Medicine, The Chinese University of Hong Kong, Shenzhen 518172, China; ^5^School of Science and Engineering, The Chinese University of Hong Kong, Shenzhen 518172, Guangdong, China.; ^6^Frontiers Science Center for Flexible Electronics, Xi'an Institute of Flexible Electronics (IFE) and Xi'an Institute of Biomedical Materials & Engineering, Northwestern Polytechnical University, Xi'an 710072, shaanxi, China.; ^7^Department of Biomedical Engineering, Chinese University of Hong Kong, Shatin, Hong Kong, China.; ^8^Monash Institute of Pharmaceutical Sciences (MIPS), Monash University, Parkville VIC 3052, Australia.; ^#^Authors contributed equally

**Keywords:** Extracellular vesicles (EVs), electrochemical detection, early disease diagnostics

## Abstract

This review article presents a detailed examination of the integral role that electrochemical detection of extracellular vesicles (EVs) plays, particularly focusing on the potential application for early disease diagnostics through EVs biomarker analysis. Through an exploration of the benefits and challenges presented by electrochemical detection vetted for protein, lipid, and nucleic acid biomarker analysis, we underscore the significance of these techniques. Evidence from recent studies renders this detection modality imperative in identifying diverse biomarkers from EVs, leading to early diagnosis of diseases such as cancer and neurodegenerative disorders. Recent advancements that have led to enhanced sensitivity, specificity and point-of-care testing (POCT) potential are elucidated, along with equipment deployed for electrochemical detection. The review concludes with a contemplation of future perspectives, recognizing the potential shifts in disease diagnostics and prognosis, necessary advances for broad adoption, and potential areas of ongoing research. The objective is to propel further investigation into this rapidly burgeoning field, thereby facilitating a potential paradigm shift in disease detection, monitoring, and treatment toward human health management.

## INTRODUCTION

Extracellular Vesicles (EVs) are lipid-bilayer-encapsulated particles secreted by various cell types into the extracellular environment, exhibiting an intricate biological character^[1-3]^. Biofluids contain a large number of EVs, which can shuttle between parental cells and other cells. Initially misunderstood as “cell dust” and a mechanism for disposing of cellular components, EVs are now recognized as abundant and stable sources of biomacromolecules including proteins, lipids, and nucleic acids, thus serving as cellular surrogates^[4-6]^. These macromolecules are instrumental in modulating various cellular processes and serve as prognosticators for disease states, including tumorigenesis, neurodegenerative conditions, and cardiovascular diseases^[7-12]^. EVs are elaborate carriers of biomolecular information, with the capacity to modify the physiology and functionality of recipient cells through the transfer of surface-bound or intraluminal molecules^[13]^. Their interaction with recipient cells can lead to significant alterations in cellular behavior, gene expression profiles, and intracellular signaling pathways-indicative of their expansive role in biological phenomena such as immunoregulatory activities, angiogenesis, and the progression of diseases^[14,15]^. EVs thereby offer a minimally invasive avenue to access and monitor pathological cells over time through serial sampling.

Nevertheless, the detection of EVs, particularly subpopulations within body fluids, is impeded by their minuscule size and low abundance, necessitating the development of more sensitive detection methodologies^[5,16,17]^. Traditional techniques, such as Western blotting, enzyme-linked immunosorbent assays (ELISAs) for proteins, and polymerase chain reaction (PCR) for nucleic acids, although widely used, require considerable sample volumes, lengthy preparation times, and the use of potentially hazardous reagents, limiting their clinical translational potential^[18,19]^. Recent advances in EV detection methods-including surface-enhanced Raman spectrometry, fluorescence labeling, and colorimetric assays-aim to address these issues by enhancing sensitivity, streamlining procedures, or mitigating costs. However, these methods often grapple with the inherent complexity of biological matrices and typically require sophisticated instrumentation and costly sample pre-treatment^[20-23]^.

In contrast, electrochemical detection platforms may offer a viable solution to these limitations, potentially enabling the rapid characterization of clinically relevant EVs for diagnostic and therapeutic applications^[6,24,25]^. Electrochemical approaches afford a multitudinous platform that not only accommodates the diversity of EVs but also achieves augmented sensitivity, rapid detection capabilities, miniaturization prospects, and affordability through strategic interfacial design and fabrication^[26,27]^. Typically, these devices employ biorecognition elements-such as enzymes, proteins, antibodies, nucleic acids, cells, aptamers, or nanobodies-that, upon specific interaction with targeted analytes, produce electrical signals commensurate with the analyte concentration^[28,29]^.

In this review, we briefly discussed the biogenesis of EVs and some disease biomarkers found in EVs, focusing on the emerging electrochemically based methods for quantitative detection of EVs. We highlight pertinent applications of EVs in the early diagnosis and monitoring of diseases, such as tumors, neurodegenerative disease, and heart disease.

## BIOGENESIS OF EVs AND DISEASE BIOMARKERS FOUND IN EVs

### Biogenesis of EVs

Recent advances have highlighted the diagnostic utility of EVs obtained from various bodily fluids, such as blood, cerebrospinal fluid, urine, and sweat. These vesicles are increasingly acknowledged as a promising biomarker source for the diagnosis of an array of diseases, functioning effectively in liquid biopsies for cancer, Alzheimer’s disease (AD), and cardiovascular conditions^[30,31]^. EV biogenesis and secretion are contingent upon a complex series of cellular events that vary with the originating cell type and the prevailing physiological or pathological states. Typically, EVs are stratified into six distinct categories: exomeres, microvesicles, exosomes, apoptotic bodies, apoptotic blebs, and argosomes; this classification is based on criteria such as vesicle size, molecular content, cellular origin, and function^[32,33]^. The differentiation of EV subpopulations, however, is impeded by the absence of specific vesicular biomarkers and their size overlap, rendering efficient subpopulation discrimination a significant challenge^[34,35]^. It is also worth noting that the term “exosomes” is frequently employed in the literature to describe a heterogeneous group of small EVs, often without conclusive evidence of their endosomal origin. Consequently, the resulting data are insufficient to ascertain their exact specificity. In the interim, they are collectively designated under the broad term “EVs”. Table 1 provides a concise compilation of these EV classifications.

**Table 1:** A summary description of the classification of EVs

**EVs**	**Size(nm)**	**Biogenesis**	**Shape**	**Function**	**References**
Exomeres	≤ 50	Unknown	Heterogeneous	Signaling and growth-promoting actions	^[36]^
Microvesicles (MVs)	100~1,000	Directly from the plasma membrane	Heterogeneous	Cellular communication, disease progression, and diagnostic instruments	^[37,38]^
Exosomes	30-150	multivesicular endosome (MVE)	Heterogeneous	Intercellular communication, diagnostic tools, drug delivery system, *etc.*	^[38,39]^
Apoptotic bodies	500-2,000	Programmed cell death	Heterogeneous	Macrophages phagocytose and clean them locally.	^[40]^
Apoptotic blebs	50-500	Increased hydrostatic pressure as a result of cellular contraction	Heterogeneous	Form the foundation of apoptotic entities with fragmented membranes.	^[33,41]^
Argosomes	/	Drosophila melanogaster wing disc cells released.	Heterogeneous	Involved in signal transduction to convey cellular information throughout development.	^[33]^

### Disease biomarkers found in EVs

EVs have increasingly gained attention as a gold mine for disease biomarkers due to their encapsulation of unique biological molecules as the “finger print” of parental cells, including proteins, lipids, and nucleic acids. The following segments delve into each class of biomarkers found in EVs^[42,43]^.

#### EVs-related proteins

Recent studies in the domain of EVs have significantly propelled the field of disease diagnostics and monitoring, particularly through the identification of protein biomarkers. These proteins are not mere passive indicators of the originating cells' biochemical status; they actively participate in modulating the physiological responses of target cells.

Prominent among the proteins secreted in EVs are enzymes, growth factors, membrane transporters, and cytoskeletal components. Their expression patterns have been correlated with a spectrum of pathologies, wherein robust associations have been established, particularly in oncology, neurology, and cardiology. Noteworthy examples include the detection of Epidermal Growth Factor Receptor (EGFR) and HER2 in EVs in the context of breast cancer^[44]^, glypican-1 (GPC-1) for pancreatic neoplasms^[45]^, a-IGF-1R in relation to lung carcinoma, claudin-4 with ovarian cancer^[46]^, and Annexin V, EpCAM, and ASGPR1 in the diagnosis of hepatic malignancies^[47]^. Furthermore, proteins derived from cerebral-origin EVs have been progressively recognized as viable biomarkers for neurodegenerative diseases, attributable to their facilitated traversal of the blood-brain barrier. Notably, EV-associated α-synuclein has been proposed as a precursor biomarker for Parkinson's disease^[48,49]^, while proteins Aβ42, T-tau, and p-tau have been implicated in Alzheimer's disease^[44]^. In the realm of cardiac health, the deployment of creatinine phosphokinase-muscle band (CPK-MB), troponin-T (TnT), and troponin I (TnI) encapsulated in EVs portends a breakthrough in the early and precise diagnosis of cardiovascular diseases^[50]^.

#### EVs-related lipids

EVs-related lipids were once considered to be stable structural components without special expression until the development of direct infusion and chromatography-mass spectrometry^[51,52]^. Numerous studies have reported the importance of the lipid composition of EVs and its influence on their mechanism of action. For example, changes in the lipidomic profile of EVs have been shown to influence the progression of various diseases, such as cancer and AD, in a manner that varies with different development stages^[53]^. EVs of different cellular origins have membranes containing various distributions of lipids. Generally, lipids in EVs could be classified into three types according to their structure:

(1) Sterols. Sterols are essential components of the lipid bilayer of cell membranes. They play several crucial roles in the structure and function of EVs, including membrane integrity, membrane fluidity, and biological function. Among them, cholesterol has been most extensively studied and is thought to be the most abundant lipids in EVs. Skotland *et al.* found the overexpression of cholesterol by quantifying 277 lipids in human prostate cancer cell-derived EVs, which may serve as biomarkers for liquid biopsy^[54]^.

(2) Glycerophospholipids. Glycerophospholipids play a critically important role in the structure and function of extracellular vesicles, such as membrane fluidity, biogenesis, and release. Phosphatidylcholine (PC), phosphatidylserine (PS), and phosphatidylethanolamine (PE) are typical glycerophospholipids. For example, the increase in glycosphingolipids has been connected with AD^[55]^, high expression of phosphatidylethanolamine is associated with lung cancer^[56]^, whereas EVs loaded with phosphatidylserine have been correlated with the inception of atherosclerosis^[57]^.

(3) Sphingolipids. Sphingolipids are integral components of cell membranes with structural and signaling roles critical to the function of EVs, such as structural role and signaling function. The current research mainly focuses on ceramide, a cell stress inducer that stimulates exosome release to regulate biological processes. Ceramide is considered to be overexpressed in cancer patients, which may be a potential biomarker in cancer diagnosis^[58]^.

#### EVs-related nucleic acids

The dawn of nucleic acid biomarkers in EVs has thrown open a fresh realm of capabilities for disease diagnosis and monitoring. Intriguingly, EVs, apart from proteins and lipids, also encapsulate both DNA and several species of RNA-messengers, ribosomal, transfer, and various non-coding RNAs, selectively packaged from the mother cell^[59]^.

The conspicuous presence of nucleic acids, particularly microRNAs (miRNAs), within EVs has been exploited for various diagnostic purposes^[60]^. Alterations in miRNA profiles have been implicated in diseases such as cancer, metabolic disorders, and cardiovascular diseases. For example, the overrepresentation of oncogenic miR-21, miR-375, and miR-27a in the EVs has been recorded as an indicator of lung and breast cancers^[61]^. In addition, some messenger RNAs (mRNAs), such as EPHA2, EGFR, and PDPN, have been commonly used for glioblastoma diagnosis^[62]^. Furthermore, an increasing number of studies have addressed the applicability of EV-associated DNA in blood for the detection of cancer-specific mutations, especially in comparison with cfDNA^[63,64]^. Meanwhile, changes in EV-related lncRNA GAS5 levels have shown considerable potential as biomarkers for diabetes^[65]^.

Overall, components like proteins, lipids, and nucleic acids identified in EVs hold substantial promise as disease biomarkers. Leveraging these promising insights in connection with the use of electrochemical detection could revolutionize our abilities to detect and diagnose diseases at their earliest stages. Future research should aim to explore this potential further, optimizing methodologies for efficient and reliable extraction and analysis of these EV constituents.

## ELECTROCHEMICAL DETECTION FOR EVs

### Classification and principles of electrochemical detection of EVs

The realm of electrochemical detection holds the promise of a transformative diagnostic approach, augmenting early detection capabilities across a spectrum of medical ailments. This modality operates on the foundation of its underlying principles, utilizing an electrode system to quantify specific analytes of interest. At its core, electrochemical detection leverages redox reactions, crucial biochemical processes mostly involving electron transfer, to generate discernible signals. Electrochemical biosensors are outfitted with bioactive elements such as nanobodies^[24]^, antibodies^[66]^, and aptamers^[67]^. Post recognition and interaction with the target, the concentration signal of the detected analytes is transformed into an electrochemical response signal, as exemplified by cyclic voltammetry (CV), electrochemical impedance spectroscopy (EIS), square wave voltammetry (SWV), and differential pulse voltammetry (DPV)^[68-70]^.

The electrochemical biosensor comprises three essential components^[28]^. The first component is the recognition element, tasked with ensuring high selectivity by selectively identifying and reacting with specific substrate molecules. The second component, the transducer, plays a pivotal role in converting the biological or chemical changes triggered by the reaction into observable physical signals that can be easily captured and processed. The third component is the data analyzer, which amplifies, outputs, and displays the detected signals. In the context of EVs diagnosis, the electrochemical EVs biosensor is tailored to specifically target EVs biomarkers such as proteins, lipids, and nucleic acids. By leveraging electrochemical principles, this biosensor converts signals related to biomarker and cellular concentrations in bodily fluids into electrical signals such as current, potential, and impedance^[71,72]^.

Compared to other diagnostic methods such as optical or molecular sensing, electrochemical detection provides distinct advantages. Notably, its capability to function in non-sterile environments and under non-sterile conditions stands out as a key strength. Additional benefits encompass the potential for compact, portable device manufacturing and heightened sensitivity, enabling the detection of minute quantities of target molecules crucial for early diagnosis. Nevertheless, challenges confront electrochemical detection, including navigating through the biological noise prevalent in testing scenarios and striking a delicate balance between specificity and sensitivity. These hurdles are presently being tackled through ongoing research and development endeavors, utilizing innovative strategies such as surface functionalization, advanced redox mediators, and the integration of nanomaterials^[73-76]^. The subsequent sections will delve into the precise utilization of electrochemical biosensors in EV analysis, focusing on key components such as EV-related proteins, lipids, and nucleic acids.

### Electrochemical biosensor for EVs-related protein

**Figure 1:**
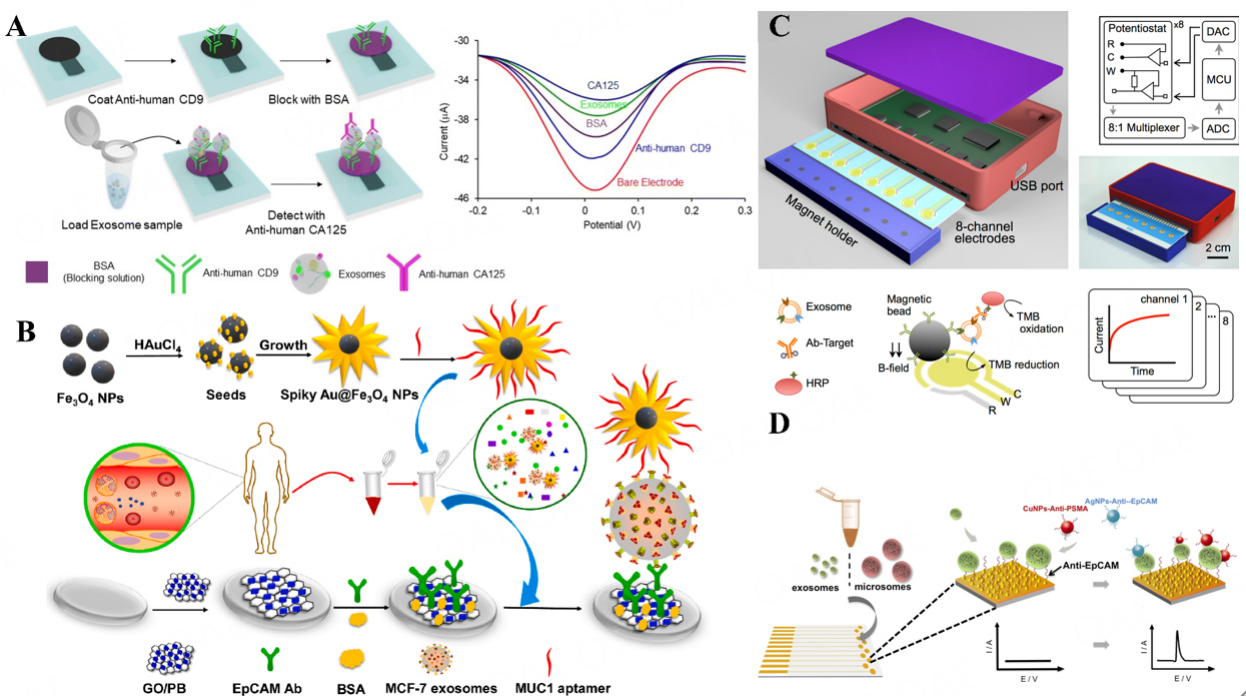
Electrochemical biosensor for detection of EVs-related protein biomarkers. (A) Electrochemical paper sandwich immunosensor: capture, quantification, and identification of generic and ovarian cancer cell-derived EVs^[18]^; (B) A sandwich-type electrochemical sensing platform for exosome enrichment and detection using Prussian blue/graphene oxide (PB/GO) and spiky Au@Fe_3_O_4_ nanoparticles^[77]^; (C) An integrated 8-channel magnetic-electrochemical exosome (iMEX) platform for profiling protein markers in EVs^[78]^; (D) A microfabricated device with gold electrodes for rapid and cost-effective multiplex analysis of EVs and microsomes^[79]^.

Multiple studies have shown the successful development of electrochemical biosensors for the detection of EVs-related proteins, especially tumor-derived EVs. As shown in Figure 1A, Kasetsirikul *et al.* developed an electrochemical paper sandwich immunosensor^[18]^. Since the CD9 protein is widely expressed on the surface of EVs, the device first captures EVs using electrodes decorated with CD9-specific antibodies. Following this, the device can identify ovarian cancer cell-derived EVs with surface-specific expression of CA125 protein, and detect as low as 7.1 × 10^8^ EVs per mL via CA125-specific antibodies, with a relative standard deviation of less than 10% (*n* = 3). Although this paper-based electrochemical technology has great potential for POCT applications due to its economical and simple characteristics, its sensitivity still needs to be further improved.

Consider existing detection methods have shortcomings such as long-time consumption and low sensitivity. Figure 1B illustrates a sandwich-type electrochemical sensing platform with an exosome enrichment strategy developed by Pan *et al.*^[77]^. This platform leverages Prussian blue/graphene oxide (PB/GO) and spiky Au@Fe_3_O_4_ nanoparticles on magnetic beads. Firstly, the MUC1 aptamer-conjugated spiky Au@Fe_3_O_4_ was used to rapidly and efficiently capture cancer-derived EVs from serum, since MUC1 protein is overexpressed in MCF-7-derived EVs. Then, GO/PB was modified on the glassy carbon electrode surface to load another EVs surface protein EpCAM antibody. The advantage of this biosensor is that the spiky Au@Fe_3_O_4_’s enrichment and signal amplification notably enhance the platform’s detection sensitivity. As a result, the proposed biosensor can detect as few as 80 EVs per μL. This proposed biosensor is an efficient tool for high sensitivity analyses of EVs, especially by greatly accelerating the time required for EVs capture to detection.

Compared with the analysis of a single specific EVs protein, which may result in high false positives, simultaneous detection of multiple specific proteins on the surface of EVs can provide more information for early diagnosis of disease. This approach allows for a more accurate representation of the molecular signature of the cells of origin, thereby enhancing the veracity of disease diagnostics and therapy monitoring. Based on this, Jeong *et al.* developed a portable assay that combines magnetic and electrochemical elements and is equipped with eight channels for swift EVs screening [Figure 1C]^[78]^. EVs were captured from patient samples by immunomagnetism and analyzed by electrochemical reaction. This biosensor had the capability to profile a variety of protein markers, including CD63, EpCAM, CD24, and CA125, all within an hour, which improves the reliability of early detection of disease. Additionally, to investigate the applicability of a nanoparticle-labeling strategy in the detection of EVs, Zhou *et al.* developed a sensor chip containing 11 individual circular gold electrodes that enables multiplexed readout. As shown in Figure 1D^[79]^, the surface of the nanostructured sensors was modified with thiolated anti-EpCAM aptamers for EVs capture, and electrochemical assay was used to detect the captured EVs by Cu and Ag nanoparticles. The advantages of this electrochemical method over traditional techniques are not only its speed, simplicity, and cost-effectiveness, but also providing more accurate information for early diagnosis of disease.

### Electrochemical biosensor for EVs-related lipid

**Figure 2:**
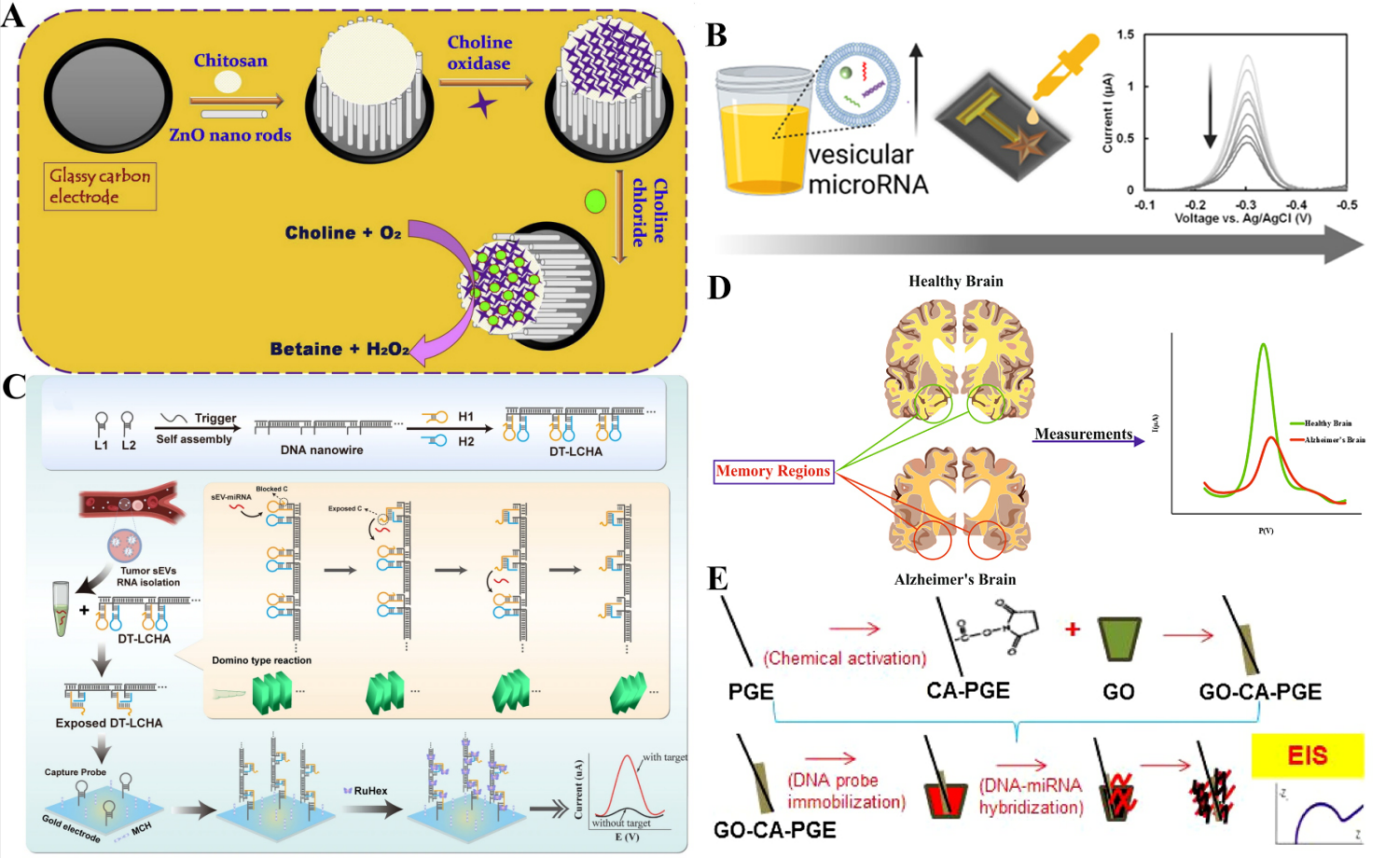
Electrochemical biosensor for detection of EVs-related lipid and microRNAs. (A) A modified glassy carbon working electrode for the development of choline biosensors^[80]^; (B) Two-step competitive hybridization assay enhances electrochemical signals for the detection of vesicular miRNA 200b in prostate cancer cell lines and human urine samples^[87]^; (C) A sensitive and rapid electrochemical biosensor for sEV-miRNA detection^[88]^; (D) An electrochemical genosensor for monitoring of miR-146a^[89]^; (E) A graphene oxide modified graphite electrode was developed for the detection of miRNA-34a-related Alzheimer’s disease and various types of cancer^[90]^.

Despite being less explored compared to protein biomarkers, lipids in EVs present an equally promising source for biomarker discovery. Fluctuations in lipid content can serve as crucial indicators of pathological states. The field of lipidomics has emerged as a key component of metabolomics, offering valuable insights into disease mechanisms. Lipids within EVs have started to surface as biomarkers in the realm of liquid biopsy. For instance, Thiagarajan *et al.* effectively distinguished breast cancer patients by assessing choline expression levels through the amperometric method, indicating potential clinical application for the sensor in this context [Figure 2A]^[80]^. Tong *et al.* developed an electrochemical biosensor for a model system of cells integrated by phosphatidylserine-modified liposome. Firstly, Annexin V was immobilized on a self-assembled layer of gold nanoparticles, which allowed stable and high loading of Annexin V on the electrode surface, offering the possibility of sensitivity enhancement. Through the specific interaction between Annexin V and phosphatidylserine, the variation of electrochemical impedance would be finally observed^[81]^.

### Electrochemical biosensor for EVs-related nucleic acid

Nucleic acid transfer within EVs can be crucial to the functioning of a multicellular organism. Current research on RNAs in EVs mainly involves miRNAs, mRNAs, and circular RNAs (circRNAs); among them, miRNAs have received the greatest attention as disease diagnosis biomarkers owing to their resistance to RNase-dependent degradation^[82-84]^. Their applications in disease diagnosis based on electrochemical biosensors will be briefly described below.

#### miRNAs

miRNAs are a tiny family of non-coding RNA molecules (22 nucleotides in length) that are a key component of EVs. Many studies have shown that abnormal miRNA regulation plays a major role in the pathological events underlying cancers and cardiac disease, as well as neurodegenerative diseases such as Parkinson’s disease (PD), Huntington's disease (HD), Multiple sclerosis (MS), and AD; thus, miRNAs could serve as potent biomarkers for AD diagnosis^[31,85,86]^.

In an innovative approach, Saha *et al.* have conceptualized a two-step competitive hybridization assay, ingeniously engineered to transduce low miRNA abundance into robust electrochemical signals. Intriguingly, the magnitude of the signal elicited is inversely proportional to the concentration of the target miRNA. With a detection threshold as low as 122 attomolar (aM), this assay has been adeptly applied to quantify vesicular miRNA 200b in prostate cancer cell cultures as well as in human urinary specimens, as depicted in Figure 2B^[87]^. Complementarily, Li *et al.* have devised an electrochemical biosensor via the integration of a DNA nanowire scaffold, which serves the dual role of a catalyst and a support structure for the hairpin assembly crucial for sensing extracellular vesicular (EV) miRNA1246, as illustrated in Figure 2C^[88]^. The assay leverages a domino-type localized catalytic hairpin assembly (DT-LCHA) for signal enhancement along three dimensions: (1) By embedding the CHA system with the DNA nanowire, it condenses the spatial gap among hairpin substrates, heightening the kinetic encounter between H1 and H2 molecules and thus facilitating a “domino effect”; (2) the DNA nanowire acts as a reservoir for a significant volume of the electroactive complex, Ruthenium Hexaamine (RuHex), which substantially escalates the resultant electrochemical readout; (3) possessing multiple DT-LCHA sites, the nanowire ensures that the immobilization of a single reactive CHA onto the capture probe exponentially amplifies the system's sensitivity, particularly for detecting scant miRNA concentrations. Through this ingenious trifecta of amplification strategies, the biosensor demonstrates an exceptional capacity to discern EV-miRNA at concentrations as minimal as 24.55 aM within a mere 20 min, while also maintaining an outstanding specificity profile. The device’s precision enables the differential diagnosis between benign and early-stage malignant gastric tumors-a testament to its potential clinic.

Khalilzadeh *et al.* have pioneered an electrochemical assay, centered on miRNA detection, to quantify miR-146a-an established biomarker indicative of neurodegenerative conditions. Within this bioassay, a capture microRNA (C-miR) is covalently linked onto a gold electrode surface, facilitating the detection of target miR-146a through Square Wave Voltammetry (SWV). The bioassay demonstrates a dynamic detection range spanning from 10 picomolar (pM) to 1 micromolar (μM), with a precision reflected by a relative standard deviation of 1.59%. Impressively, the lower limit of detection stands at 10 pM. This method typifies a more accurate and sensitive approach for the early prognosis of neurodegenerative diseases such as Alzheimer's and Parkinson's diseases, as depicted in Figure 2D^[89]^. In parallel, Congur *et al.* conceived an impedimetric biosensor using Graphene oxide (GO)-modified graphite electrodes for the meticulous and selective detection of miRNA-34a, a notable biomarker for Alzheimer's disease. The optimization of this platform resulted in detection limits of 261.7 nanoMolar (nM) in phosphate-buffered saline (PBS, pH 7.4) and an enhanced sensitivity of 72.5 nM within a PBS matrix supplemented with 50% fetal bovine serum (FBS), demonstrating its potential utility in complex biological samples [Figure 2E]^[90]^.

#### mRNAs

mRNAs are coding RNA molecules synthesized through a meticulously regulated process involving transcription, intron splicing, and 3’ end modification, orchestrated by RNA polymerase II utilizing a single-stranded DNA template^[91]^. Once transcribed, mature mRNAs are selectively incorporated into EVs, which then ferry these informational molecules to target cells, dictating the subsequent translation into proteins. Hence, mRNAs harbored within EVs serve not only as mirrors reflecting the physiological spectrum of the progenitor cells but also as regulatory agents influencing the biological processes in recipient cells. The expression of specific mRNAs in EVs has consequently garnered substantial attention as salient biomarkers for clinical diagnostics^[92]^.

**Figure 3:**
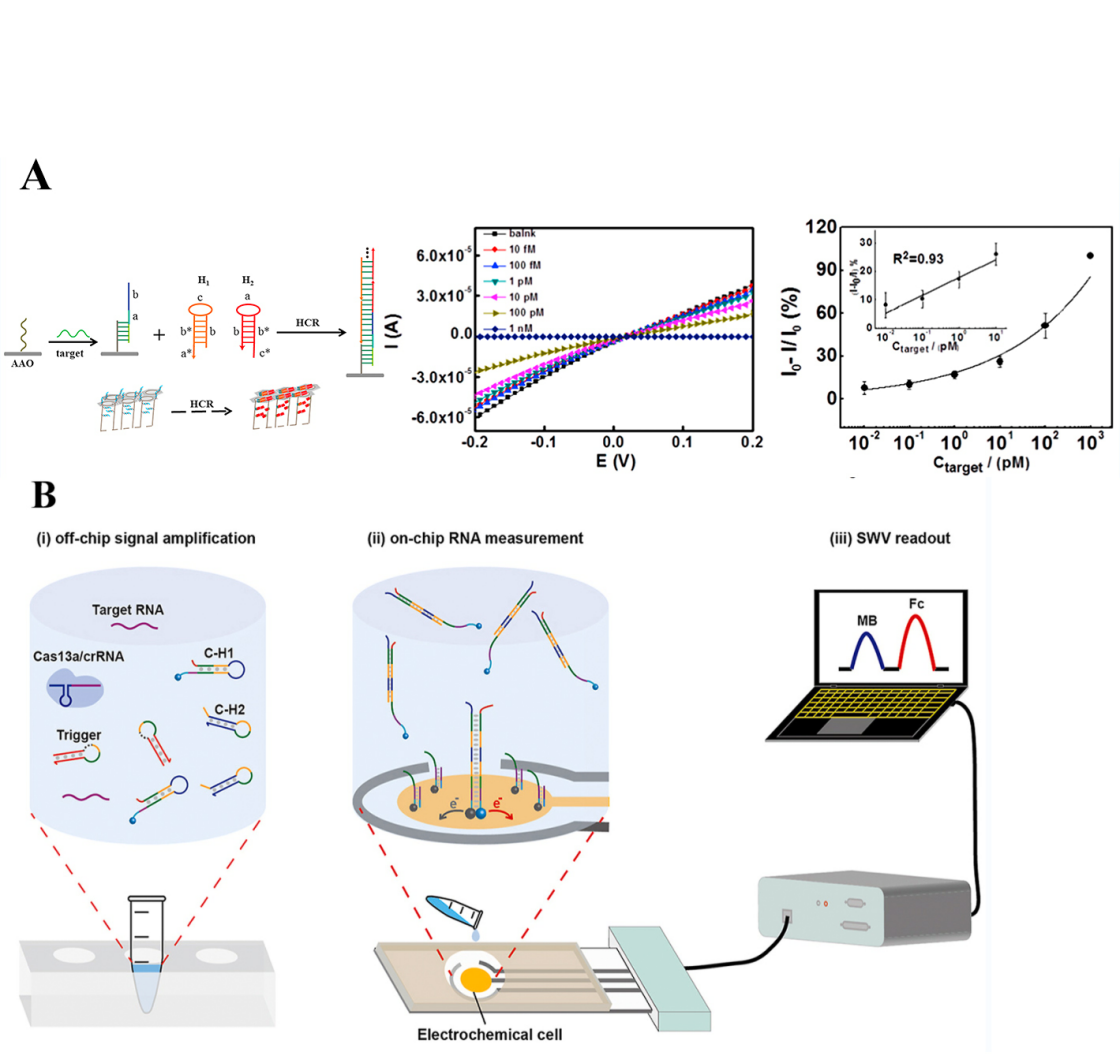
(A) A nanopore biosensor for sensitive and label-free nucleic acid detection based on hybridization chain reaction amplification^[93]^; (B) A CRISPR/Cas13a-powered catalytic electrochemical platform for mRNA diagnostics^[94]^. *: complementary strand

Zhao *et al.* reported the development of a sophisticated nanopore electrochemical biosensor, incorporating a hybridization chain reaction (HCR) for signal amplification, targeting the detection of low-abundance survivin mRNA-a marker commonly overexpressed in various cancer cells-for the facilitation of early oncological diagnosis. The operation of nanopore-based sensors is predicated on a simple yet effective principle: the selective translocation or adsorption of target molecules within or onto the nanopores alters the ionic current, rendering these changes quantifiable. This particular biosensing approach achieves significant sensitivity, detecting concentrations as low as 30 femtomolar (fM) and displaying a linear dynamic range between 0.1 and 10 pM. The method has also demonstrated robust capabilities for the analysis of specimens derived from real-world samples, as elucidated in Figure 3A^[93]^.

Additionally, Sheng *et al.* meticulously engineered a dual isothermal amplified electrochemical biosensor capable of identifying TTF-1 mRNA, a marker pertinent to non-small cell lung cancer. This biosensor integrates Cross-Linked Hybridization Chain Displacement (CHDC) with the CRISPR/Cas13a system, achieving a remarkably low detection limit of 50 attomolar (aM) with a swift analytical readout achievable within six minutes, requiring a mere 10 μL of sample volume, as elucidated in Figure 3B^[94]^. Nonetheless, the application of mRNA as a biomarker is constrained by challenges such as their inherently low abundance in biological samples, their relatively short half-life, and the diversity of mRNA species. It is, therefore, imperative to pursue the development of electrochemical methods possessing ultra-sensitive detection capabilities for mRNA, in order to unlock the full diagnostic potential that these biomolecules may offer.

#### circRNAs

**Figure 4:**
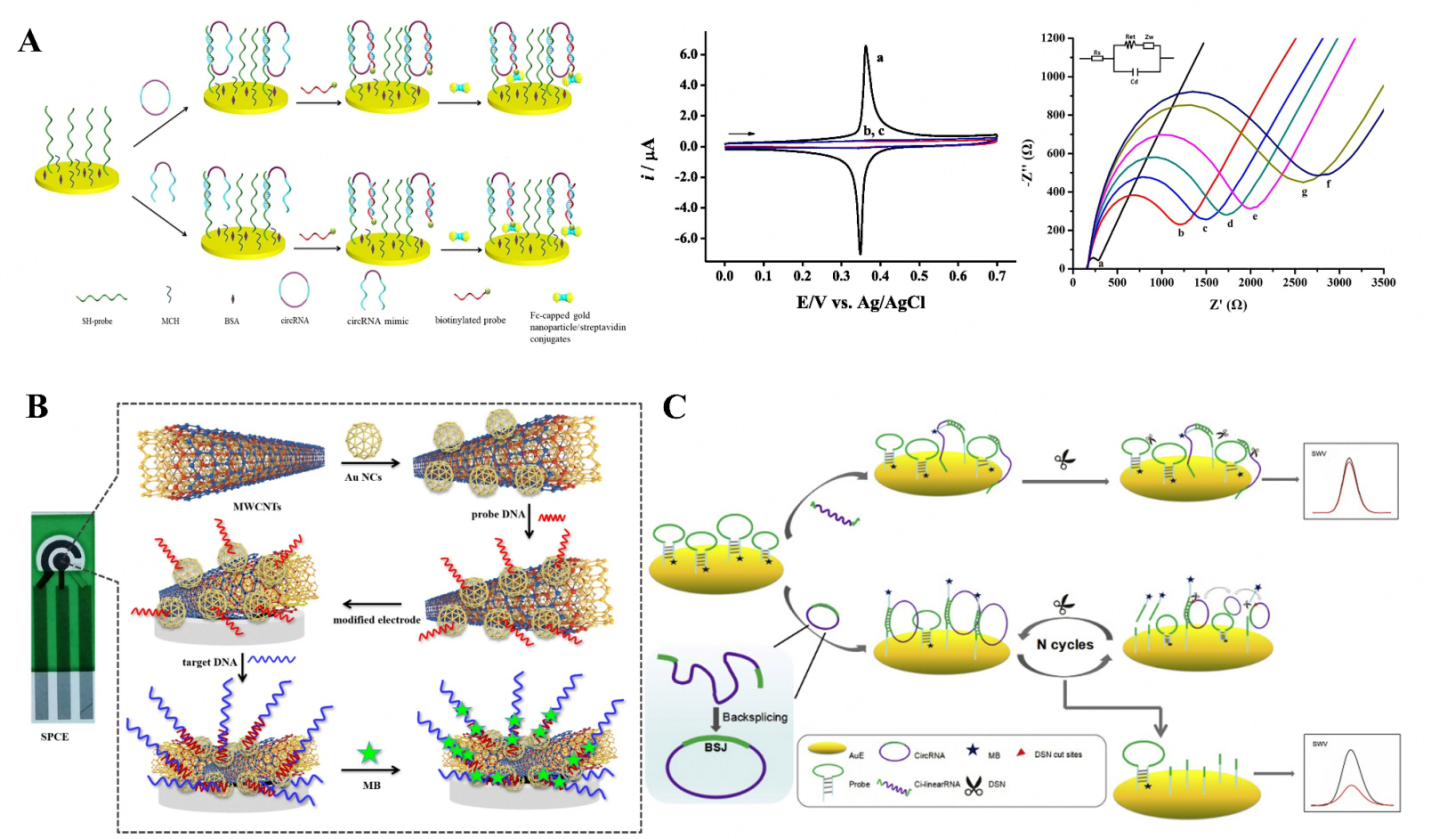
Electrochemical biosensor detects EVs-related biomarkers of different diseases. (A) Amplified electrochemical detection of circular RNA in breast cancer patients using ferrocene-capped gold nanoparticle/streptavidin conjugates^[96]^; (B) A biosensor for the detection of the lncRNA biomarker MALAT1 in non-small cell lung cancer^[97]^; (C) An electrochemical platform based on the combination of back-splice junction and duplex-specific nuclease for the detection of circRNAs^[98]^.

circRNAs are non-coding RNAs, which were recently discovered in EVs. Current studies have shown that circRNAs could efficiently resist exonuclease-mediated destruction as circRNA bases are interconnected to form a covalent closed-loop structure^[42]^. From this point of view, circRNAs hold the promise of becoming a stable biomarker for others. In addition, several studies have shown that circRNAs are more stable than mRNAs and miRNAs, and evidence from recent studies discovered that circRNAs are dysregulated in cancer, implying that they may be useful as biomarkers in cancer diagnosis. Therefore, circRNAs are thought to be another novel biomarker for early disease diagnosis^[95]^. Electrochemical biosensing based on circRNAs has been reported. For example, Wu *et al.*, developed a sandwich structure with two complementary DNA probes that were designed to recognize target circRNAs as a diagnosis of triple-negative breast cancer. The biotinylated probe was attached to ferrocene (Fc)-capped gold nanoparticle/streptavidin conjugates for voltammetry of circRNAs. The Fc oxidation current was proportional to circRNA concentration in the range of 0.5 and 10 pM, with a detection limit of 0.22 pM [Figure 4A]^[96]^. Chen *et al.* successfully designed a novel effective and ultrasensitive electrochemical biosensor for the screening of non-small cell lung cancer by non-coding RNAs detection by a gold nanocage coupled with an amidated multi-walled carbon nanotube (Au NCs/MWCNT-NH2)-decorated screen-printed carbon electrode (SPCE). This biosensor has a large surface area, superior conductivity, and excellent biocompatibility and shows a wide linear range (10^-7^-10^-14^ M) and a low detection limit (42.8 fM) [Figure 4B]^[97]^. Jiao *et al.* designed a simple electrochemical approach for detecting circRNAs. Firstly, circRNAs were identified by back-splice junction (BSJ) located in hairpin probes. Then, the substrate length is selected by duplex-specific nuclease (DSN) to trigger DSN-assisted signal amplification, thereby boosting sensitivity. As a result, this approach for circRNA detection has a detection limit as low as 3.47 fM, owing to improved selectivity, reproducibility, and stability. This approach has also been used to detect circRNAs in human serum samples, which has the potential to be very useful in clinical diagnosis applications [Figure 4C]^[98]^.

## CONCLUSIONS AND FUTURE PERSPECTIVES

Electrochemical methodologies employed in the identification and analysis of biomarkers within EVs signify a promising frontier, augmenting the precision of disease diagnostics and prognostics. These methodologies facilitate the quantification of proteinaceous, lipidic, and nucleic acid markers down to infinitesimal concentrations, thus endorsing the expeditious detection of pathologies, inclusive of, but not restricted to, carcinomas and neurodegenerative afflictions. Given that EVs mirror the cytophysiological state of their source cells, they have an unparalleled potential to yield pivotal prognostic insights. The systematic integration of these detection modalities into EV isolation and analytic protocols may herald a transformative epoch in personalized healthcare modalities. Notwithstanding, for a more ubiquitous adoption, these modalities necessitate several advancements: diminution in production expenditures, acceleration of analytic throughput post-incubation, and a simplification of the operational complexity to diminish the requirement for specialized technical acumen. Additionally, refinement in the sensitivity and specificity of both capture mechanisms and analytic procedures is paramount. The aspiration for a reliable electrochemical biosensor capable of single-entity sensitivity is imperative, particularly for the discernment of scarcely abundant EV subpopulations or the intricately altered proteomic constituents-post-translational modifications (PTMs) in EV proteins-where current modalities remain wanting. Uniform standardization in EV isolation processes, biosensor design, and biomarker corroboration also remains crucial.

While the utility of sizeable sample volumes to formulate disease correlations through high-throughput, multiplexed electrochemical sensors remains in a nascent developmental phase, it underscores a landscape rich with opportunity. Concurrently, the evaluation of EV-associated metabolites has emerged as a promising domain within the detection and diagnostic arena, meriting extensive investigation into electrochemical biosensors attuned to additional EV-related metabolomic variants^[99-101]^. In summary, electrochemical biosensors represent an incipient yet rapidly evolving technology poised to redefine thresholds in healthcare, environmental surveillance, and biosecurity. Imminently, these instruments are poised for further advancements via the integration of nanotechnology and avant-garde signal transduction frameworks, thereby amplifying their diagnostic acumen. Such progress intimates the prospect of real-time, bedside diagnostic assays and, by extension, the facilitation of timely, customized medical interventions.

## DECLARATIONS

### Copyright

© The Author(s) 2024.
